# Foraging preferences of dairy cows grazing on contrasted multispecies swards

**DOI:** 10.1016/j.vas.2025.100439

**Published:** 2025-03-03

**Authors:** Mira Hesselmann, Sarah Thorne, Amarante Vitra, Andrea K. Steiner, Florian Leiber, Marie T. Dittmann

**Affiliations:** Department of Livestock Science, Research Institute of Organic Agriculture FiBL, Ackerstrasse 113, 5070, Frick, Switzerland

**Keywords:** Feeding behaviour, Dietary choice, Pasture, Botanical biodiversity, Plant secondary compound, Ruminant

## Abstract

Selective foraging has been reported in many wild and domesticated ruminants, yet modern livestock production systems rarely allow animals to express this natural behaviour. However, it is assumed that giving livestock the opportunity for forage selection positively influences their health and welfare. This study investigated the forage selection of individual dairy cows on pasture. Over one grazing season, a herd of 23 dairy cows was observed grazing on an experimental pasture in Switzerland, which consisted of different plant mixtures. The cows had unrestricted access to all mixtures and their individual foraging behaviour was documented by scan sampling. The four mixtures were rich in grasses (G), grasses and legumes (L), grasses and tanniferous plants (T), and grasses and herbs containing essential oils (E). The results revealed a partial preference for the L mixture at herd level across all grazing rotations (*P* ≤ 0.001). All mixtures were frequented, indicating that the herd utilized the array of available plants. Individual preferences varied and not all cows followed the herd's average pattern. Individual preferences shifted throughout the grazing season, but the pattern of this variability differed between animals. The findings demonstrate that dairy cows actively select from different pasture plants and that preferences vary between individuals and over time. Importantly, average herd preferences do not accurately represent the behaviour of all cows. Individual preferences should be considered when aiming to meet the foraging needs of all herd members.

## Highlights

• Dairy cows were observed foraging on four distinguished multispecies swards.
• On herd level, the cows preferred the mixture rich in legumes.
• Preferences of individual cows deviated from the herd average.
• In most individuals, preferences changed throughout the grazing season.

## 1 Introduction

In modern intensive grazing production systems, pastures are designed to supply the animals with a homogenous diet, often comprising a limited number of plant species, such as ryegrass and clover. Such pastures do not allow ruminants to express a behaviour natural to most animal species: the selection of specific feeds or feed components, which has been reported in various domestic and non-domestic ruminant species (Engel, 2003; Rutter et al., 2004; J. J. Villalba et al., 2006a). Feed selection seems to be much more complex than initially stated by optimal foraging theory (OFT), which proposes that animals select their feed according to the maximum energy yield to improve their ‘fitness’ (Newman et al., 1995). It has been shown that the foraging behaviour of ruminants under semi-natural conditions also includes elements such as species-specific spatial foraging behaviour (Leiber et al., 2009), foraging for less abundant plants (Inglis et al., 1997), or selecting plants containing bioactive secondary plant compounds (Villalba et al., 2010). It has been suggested that selective consumption of plants with certain plant secondary metabolites (PSM) could be a behavioural mechanism to positively influence digestive efficiency and metabolic health (Leiber et al., 2020). The consumption of plants containing essential oils, phenols, or alkaloids can help to control the microflora in the rumen and thus enable optimal nutrient uptake and prevent undesirable foregut fermentation processes, such as bloat, acidosis or excessive ammonia formation (Goel et al., 2008; Kapp-Bitter et al., 2023; Snelling et al., 2019; Vasta et al., 2019). The health status of the animal also appears to affect feed choice, since the avoidance or intentional consumption of plants with certain toxins may help control diseases or parasites, as shown in studies on self-medication of ruminants (Poli et al., 2018; Villalba et al., 2006b, 2010). For instance, sheep experiencing physical discomfort selectively consumed certain PSMs, and those heavily infected with nematodes ingested more tannins (Villalba et al., 2006b, 2010). Other studies have shown that PSMs, such as alkaloids, terpenes, and phenolics aid in parasite control (Rochfort et al., 2008), improve protein absorption (Min & Hart, 2003) or help control of the fungal and bacterial flora in the digestive tract (Villalba & Provenza, 2007). The anthelmintic effect of certain bioactive compounds in forbs/herbs has been demonstrated in sheep (Lisonbee et al., 2009; Marley et al., 2006), goats (Amit et al., 2013) and cattle (Desrues et al., 2016). Additionally, external factors influence the foraging behaviour of ruminants. Cows and sheep adjust their preference for certain plants throughout the day (Rutter, 2010; Rutter et al., 2004) and seasonal changes influence grazing behavior (Westoby, 1978). For example, dairy heifers exhibit seasonal variations in grazing time, bite number, and bite rate on different grass species (Soder et al., 2022).

Despite evidence of selective foraging, modern intensive grazing systems rarely allow ruminants to express this behavior. Livestock are often fed mixed rations that meet the nutritional needs of the average herd member (Moscovici Joubran et al., 2021; Rutter, 2010), lack variety in sensory characteristics, nutrient content, and PSMs. This restriction may result in frustration and reduced animal welfare (Beck & Gregorini, 2020; Leiber et al., 2020; Rutter, 2010; Villalba et al., 2010).

One option to enable ruminants to express forage choice is to increase pasture plant diversity through the establishment of multispecies swards. Multispecies swards offer numerous benefits, including medicinal effects (Alomar et al., 2018; Grace et al., 2019; Peña-Espinoza et al., 2016; Wilson et al., 2020), a broader range of nutrients and bioactive secondary metabolites (Cranston et al., 2015; Hopkins, 2004), different palatability of vegetation (C/N-ratio) (Whitehead, 2000), and a psychological stimulation through sensory variety. They can also enhance animal performance (McCarthy et al., 2020; Roca-Fernández et al., 2016), promote soil carbon sequestration (Cong et al., 2014; O'Mara, 2012; Skinner & Dell, 2016), incrase plant biomass production (Finn et al., 2013; Nyfeler et al., 2009), reduce nitrogen leaching (Bryant et al., 2017), increase biodiversity (Haddad et al., 2001; Wan et al., 2020), and improve drought resilience (Isbell et al., 2015; Komainda et al., 2020; Lüscher et al., 2022; Vogel et al., 2012).

Previous studies on forage selection in domestic ruminants have mainly focused on herd-level patterns or breed comparisons (Hessle et al., 2014; C.M. Pauler et al., 2020a), with little attention to individual feeding choices. One study in housed cattle demonstrated that certain individuals spent more time eating single, novel feed components than other animals which preferred the usual homogeneous feed ration (Meagher et al., 2017). Since targeted feed selection may be influenced by physiological factors such as milk yield, nutritional status, personality, or rumen milieu, understanding individual preferences is essential for meeting each animal's needs. The present study aimed to investigate how cows behave when offered a choice of different multispecies swards consisting of a variety of forage plants with different active ingredients. Throughout one grazing season, the foraging behaviour of a dairy herd was observed on an experimental pasture containing four swards differing in botanical compositions, nutrient content and PSM levels.

The study tested three hypotheses: 1) At herd level, cows spend more time grazing on certain plant mixtures than on others. 2) Individual cows display distinct preferences that may differ from the herd average. 3) These preferences remain consistent throughout the grazing season.

## 2 Material and methods

### 2.1 Experimental site

**Fig. 1:**
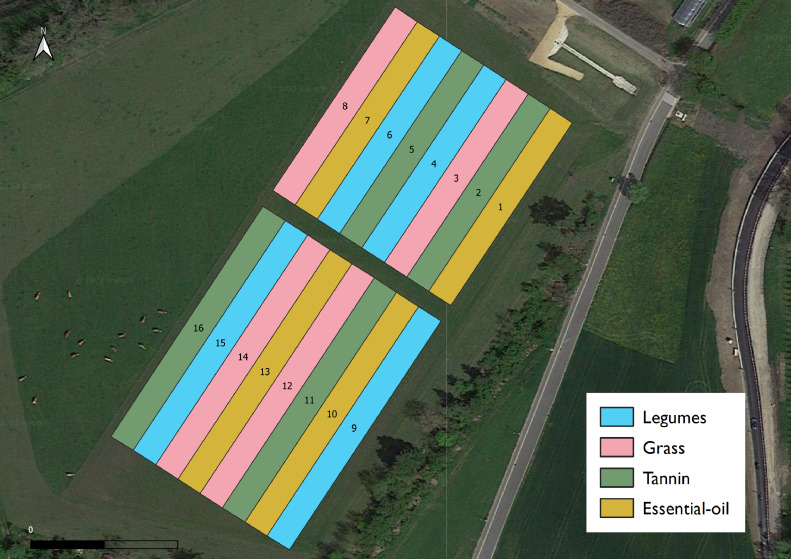
Map of the experimental site. The pasture was split into two blocks of eight plots each. Each of the four botanical treatments was replicated twice per block. During each grazing rotation, cows had daily alternating access to each block, where they had free access to each of the eight plots.

During one grazing season (April to October), a herd of dairy cows was observed while grazing on an experimental pasture consisting of 16 plots with four different plant mixtures (Fig. 1). The experimental pasture covered a total area of 1.2 ha on the FiBL farm, in Frick, Switzerland (altitude 352 m). The swards were established two years prior to the observed grazing season and consisted of four different plant mixtures established in four plots each. All mixtures contained grasses and varying amounts of legumes and forbs. The mixtures were distinguished by the following properties: rich in grasses (G), rich in grasses and legumes (L), rich in grasses and plants that contain high concentrations of tannins (T), and rich in grasses and herbs that contain essential oils (E) (Table 1). Two months before the beginning of the trial, the plots rich in grasses (G) were fertilized with an organic fertilizer (LANDOR N-BIO 12 %) at a rate of approximately 100 kg N per ha.

**Table 1:** Botanical and nutrient composition of the four plant mixtures on the experimental pasture. Values are averaged for the four replicates over six rotations (mean ± SD). The DM yield is summed up over the entire grazing season from April to October. The species listed in the rows only include those occurring >1%.

	Essential oil	Grass	Legume	Tannin
Grasses* %	31.5 ± 17.4	58.1 ± 21.3	26.4 ± 10.9	17.1 ± 13.2
**Legumes** %	46.3 ± 17.6	27.5 ± 18.9	68.5 ± 12.2	38.3 ± 15.1
*Trifolium repens* %	45.3 ± 17.7	26.6 ± 18.9	35.6 ± 15.9	22.3 ± 14.4
*Trifolium pratense* %	0.6 ± 2.9	0.9 ± 4.8	*27.6±12.6*	0.7 ± 2.9
*Medicago sativa* %	0.0 ± 0.0	0.0 ± 0.0	5.3 ± 9.2	0.0 ± 0.0
*Lotus corniculatus %*	0.1 ± 1.3	0.0 ± 0.0	0.0 ± 0.0	15.2 ± 15.1
**Forbs** %	22.3 ± 14.9	14.4 ± 13.1	5.1 ± 6.6	44.6 ± 13.6
*Taraxacum officinale* %	10.8 ± 10.8	9.3 ± 9.6	3.2 ± 5.5	4.2 ± 5.2
*Convolvuslus arvensis* %	1.1 ± 2.9	3.5 ± 6.2	1.4 ± 2.8	0.3 ± 1.0
*Plantago lanceolata* %	0.2 ± 1.3	0.2 ± 1.6	0.0 ± 0.5	12.1 ± 8.2
*Cichorium* sp. %	0.1 ± 0.6	1.1 ± 5.5	0.1 ± 1.2	27.2 ± 15.0
*Achilea millefolium* %	7.9 ± 8.6	0.0 ± 0.0	0.0 ± 0.0	0.0 ± 0.0
*Carum carvi* %	1.4 ± 1.8	0.0 ± 0.0	0.0 ± 0.0	0.0 ± 0.0
**Dicot species seeded but occurring at** <**1 %**	*Origanum vulgare* *Thymus* sp.	*–*	*–*	*Sanguisorba minor*
DM yield (t/ha)	7.93 ± 0.61	8.00 ± 0.72	9.04 ± 0.41	6.95 ± 0.37
OM (g/100*g* )	89.0 ± 0.9	89.1 ± 1.2	89.2 ± 0.9	88.1 ± 1.8
CP (g/100*g* )	19.1 ± 2.0	17.9 ± 1.7	21.7 ± 0.7	17.6 ± 1.4
NDF (g/100*g* )	39.0 ± 2.6	44.1 ± 3.1	38.2 ± 2.5	37.8 ± 3,8
ADF (g/100*g* )	26.6 ± 3.2	27.6 ± 2.9	25.0 ± 3.8	27.5 ± 4.6
ADL (g/100*g* )	4.5 ± 1.5	4.0 ± 1.9	4.5 ± 1.3	5.9 ± 1.8
CF (g/100*g* )	4.0 ± 0.4	4.1 ± 0.5	4.4 ± 0.3	3.7 ± 0.2
NEL (MJ/kg)	6.1 ± 0.2	5.9 ± 0.2	6.3 ± 0.4	5.8 ± 0.2
TEP (g/100*g* )	1.95 ± 0.03	1.94 ± 0.08	2.02 ± 0.08	2.31 ± 0.16
CT (g/100*g* )	0.45 ± 0.05	0.32 ± 0.02	0.38 ± 0.02	0.89 ± 0.26

DM dry matter, OM organic matter, CP crude protein, NDF neutral detergent fibre, ADF acid detergent fibre, ADL acid detergent lignin, CF crude fat, NEL net energy lactation, TEP total extractable phenolics, CT condensed tannins.

*Grass species, which were not distinguished during botanical assessments, comprised *Festuca rubra, Festuca arundinacea, Poa pratensis* and *Lolium perenne.*

The experimental pasture was divided into 2 paddocks bounded by electrical fencing, further referred to as blocks. Each block consisted of 8 plots (with 2 replicates of each mixture). The Northern block had a size of 5400 m^2^ while the South block measured 6768 m^2^. Each plot had a width of 9 m and a length of 75 m in the North block and 94 m in the South Block. The Northern and Southern blocks were grazed in daily alternation during the grazing cycles. The average stocking density amounted to 18 LU/ha (≙ 9 t live weight/ha) for the North block and 14 LU/ha (≙ 7 t live weight/ha) for the South block. Within each block, the cows could move freely and had access to all four mixtures. To ensure that the vegetation was high enough to provide all individuals with the option to graze on all mixtures, rising plate meter measurements were performed on all plots after each day. The vegetation was not grazed below an average RPM height of 10 cm in any of the plots. After each grazing rotation, all plots where topped to 8 cm to ensure an even regrowth of the vegetation. The pasture was then rested between 2.5 and 4 weeks before the next grazing cycle. During this time, the herd grazed on other pastures.

### 2.2 Animals

The experimental herd comprised 23 Swiss Fleckvieh dairy cows, which were housed on the FiBL farm in Frick, Switzerland. At the start of the experiment in April 2022, the cows’ age ranged between 2 and 8 years (mean: 4) and they were on average at 140 ± 64 DIM (days in milk; range: 0 - 289). Over the grazing season, lactation numbers ranged from 0 to 5 and the cows’ body condition score (BCS, based on Isensee et al., 2014) from 2.75 to 3.75 (mean: 3.25). The average milk yield recorded by the milking robot amounted to 12 kg/*d* ± 4.6 with a range of 3–19. Newborn calves were allowed to nurse from their mothers for at least two weeks after birth and this milk loss was not accounted for in the recorded yield. During the study, 13 cows transitioned from dry to lactating. On a few occasions, cows that had recently given birth were kept in the calving box and were excluded from the observations. From rotation 3 to 6 a bull was present in the herd.

The farm was Demeter certified and followed an extensive feeding regime: cattle were fed ad libitum with hay and small amounts of clover-grass silage in the barn. Lactating cows additionally received 0.5–2.5 kg/d of parlor feed (Mühlennachproduktgemisch aus “Krüsch”, Steiner Mühle AG, Zollbrück im Emmental, Switzerland) in the milking robot. During the grazing season, fresh forage was available on pasture. If the animals did not graze on the experimental site, they had daily access to other pastures. These were characterized by common grassland species such as *Trifolium repens, Trifolium pratense, Plantago lanceolata, Potentilla reptans, Taraxacum* spp., *Ranunculus acris, Onobrychis viciifolia, Lotus corniculatus* and grass species such as *Lolium perenne, Poa pratensis, Festuca arundinacea*, and *Festuca rubra*. Thus, the animals were already exposed to several species present in the experimental pasture. All cows had collars with number tags, which, in addition to coat colour and pattern, facilitated their identification.

### 2.3 Behavioural observations

To minimize the effect of the observer on the behaviour of the cows, the cows were habituated to the presence of the observers on pasture for two weeks before data collection. The foraging behaviour of the herd was observed during 6 grazing cycles, each consisting of 6 to 8 consecutive days, during which the cows grazed on the experimental pasture. During this time, the cows spent 1.5 h to 6 h per day on pasture, at different times of day (e.g. morning, noon, afternoon, and evening). The recording of behavioural observations began 10 min after the animals were let out onto the experimental pasture. The observations consisted of scan sampling at 10-minute intervals, recording the location (plot number), body position (standing, lying, walking), and behaviour (foraging, drinking, ruminating, social interaction, no visible jaw activity) of each cow. Behaviours were defined based on an ethogram adapted from Hessle et al. (2014). The cows were observed until the farmer opened the gate to let them into the stall at a previously agreed time. For each animal, the observational protocol resulted in 750 to 860 scans. Observations were carried out by three observers (MH, MD, ST), who had been trained together in using the observational protocol during a pre-study phase of 9 days before the beginning of the experiment.

### 2.4 Botanical assessments and nutrient composition

One to two days before each grazing cycle, the botanical composition of each plot was assessed and samples were taken for nutrient analysis from six randomly allocated 50 × 50 cm squares per plot. The botanical assessment followed the method described in Klapp (1965). The plant material in each quadrat was then cut to a height of 3 - 4 cm with electrical shears and collected. Samples were oven-dried at 60 °C for 24 h. The dried samples were milled to 1 mm using the mill SM 100 (Retsch GmbH, Haan, Germany). After the determination of dry matter (DM), nutrient analyses were performed by an external laboratory. Analyses included the determination of the contents of organic matter (OM), crude protein (CP), acid detergent fiber (ADF), acid detergent lignin (ADL), neutral detergent fiber (NDF), crude fat (CF), total extractable phenolics (TEP) and condensed tannins (CT). These analyses were carried out as described in Kapp-Bitter et al. (2023). Net energy for lactation (NEL) was calculated according to Daccord et al. (2017).

### 2.5 Data analysis

Before analysis, a dataset was created including only scans where cows had been observed foraging. Datapoints where cows were ruminating, grooming, walking around or expressing other behaviours were excluded from analysis. Based on this dataset, it was calculated, which proportion of scans, on average, each of the cows spent foraging in each of the mixtures within each rotation. These individual averages within each rotation were used to calculate what proportion of scans was spent feeding in each mixture on herd level. All statistical analyses were performed using R Studio (R version 1.4.1717). To investigate whether the average proportion of scans in which cows were observed foraging differed between the four plant mixtures at the herd level, we applied a linear mixed model using the lmerTest package in R (R version 1.4.1717). In this model, the response variable was the average proportion of scans spent foraging per cow per rotation. The fixed effect included the plant mixture (four levels), while cow ID was included as a random effect to account for repeated measures within individuals across rotations. In addition to the herd-level analysis, individual-level preferences and changes across the six grazing rotations were explored. For this purpose, separate linear models were applied for each cow using the full dataset of all scans. The response variable was the proportion of scans observed foraging, with plant mixture and rotation number as fixed effects. These models were run separately for each cow to capture individual foraging preferences and their potential development over time.

To determine if the proportions of scans which each individual cow spent foraging in each of the mixtures within each rotation deviated significantly from an equal distribution (i.e. spending 25 % of the scans foraging in each mixture), Chi-square tests were performed. P-values were corrected for multiple testing after Benjamini-Hochberg as described in Victor et al. (2011). The false discovery rate was set to 5 %. The adjusted threshold value for statistical significance obtained by the P-value correction after Benjamini-Hochberg was about *P* = 0.03. Therefore, all P-values < 0.03 were assumed to be statistically significant. Preferences were defined as A) the mixture the herd or the individual cow spent the highest proportion of scans foraging in, provided B) the results of the mixed or linear model or the Chi-square-test (correction after Benjamini-Hochberg) revealed that the proportion of scans spent foraging in the four mixtures deviated significantly from an equal distribution. Some individuals spent an equally high proportion of scans foraging in two mixtures in certain rotations. In these cases, it was defined that the animal showed no clear preference.

To assess the temporal variability in the proportions of scans the cows spent foraging in the different mixtures, coefficients of variance (cVar) were calculated. The cVars were calculated based on the average proportion of scans each cow spent in each mixture for each rotation (*n* = 6 for each cow in each mixture).

cVar = (Standard deviation / Proportion of observations per individual in each mixture)*100

The cVars indicate the extent of variance of individual preference between rotations. Low cVar values indicate a consistent foraging behavior of an individual in one plant mixture, high cVar values indicate inconsistent foraging behavior of an individual in a mixture. As there is no global definition on thresholds for cVar values indicating low or high consistency, these values can only be applied to compare individuals with each other.

## 3 Results

The botanical and nutrient composition of the four different plant mixtures is shown in Table 1. Overall, the four mixtures were distinct in terms of nutrient contents and the proportion in which plant species occurred in them. Some species, which had not been sown (*Taraxacum officinale, Convolvuslus arvensis*) established themselves at a considerable proportion, but overall, the mixtures were distinct in the desired characteristics: G had the highest proportion in grasses and the highest NDF content, L had the highest proportion in legumes, the highest protein content and the highest NEL values, T had the highest proportion of dicot species rich in tannin (*Lotus corniculatus, Plantago lanceolata, Cichorium* sp.) and the highest levels in phenolics and tannins, E had the highest proportion of forbs rich in essential oils (*Achilea millefolium, Carum carvi*).

### 3.1 Foraging preferences on herd level

**Fig. 2:**
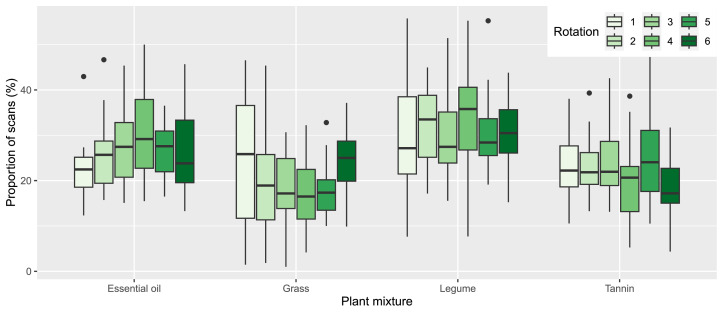
Proportions of scans individual cows were observed foraging in each of the plant mixtures. Each boxplot is based on the averages of all cows present on pasture during the respective rotation.

On average over all rotations, the cows spent 31 ± 9 % of the scans foraging in mixture L, 26 ± 8 % in mixture E, 22 ± 8 % in mixture T and 20 ± 10 % in mixture G. The proportion of scans the cows spent foraging in the four mixtures differed significantly between mixtures (*P* < 0.001), except between the mixture T and G (*P* = 0.10) (Fig. 2). There was no significant linear effect of rotation on the proportions spent foraging in the different mixtures. In all rotations, the herd spent the largest proportion of scans foraging in mixture L.

### 3.2 Preferences of individual animals

All cows spent time foraging in all four mixtures and therefore expressed only partial preferences for certain mixtures. Averaged over all rotations, 12 cows spent the highest proportion of scans feeding in the L mixture, seven cows in E, two cows in G and two cows in T (Table 2). Over all rotations, 17 cows showed significant differences (*P* < 0.05) between the proportions of scans spent foraging in the four mixtures. In six cows, the average proportions over all rotations did not differ significantly between mixtures.

**Table 2:** Mean (± SD) proportions of scans the cows were observed foraging in the respective plant mixture averaged over the six rotations. For each mixture, the respective Coefficients of Variance (cVars) are shown. Low cVars indicate a high consistency of the individual between rotations in the respective plant mixture, and high proportions a low consistency between rotations. Different superscript letters indicate significant differences in mean proportions between plant mixes based on linear models including plant mixture and rotation as fixed effects.

Cow ID	Essential oil		Grass		Legume		Tannin		Mixture the cow spent the highest proportion of scans foraging in
	**Mean %**	**cVar**	**Mean %**	**cVar**	**Mean %**	**cVar**	**Mean %**	**cVar**	
#1	28.9 ± 5.4	18.1	24.6 ± 10.7	38.6	26.7 ± 11.5	40.8	19.7 ± 3.6	18.8	Essential oil
#2	29.4 ± 8.5 ^A^	27.6	20.3 ± 5.6 ^B^	25.1	32.9 ± 8.0 ^A^	25.2	17.3 ± 6.3 ^B^	33.7	Legume
#3	26.0 ± 2.1 ^A^	8.6	12.1 ± 4.7 ^B^	38.8	37.1 ± 5.5 ^C^	14.8	24.9 ± 5.8 ^A^	24.0	Legume
#4	18.8 ± 5.9 ^A^	28.9	33.1 ± 7.9 ^B^	25.2	27.2 ± 8.1 ^AB^	29.1	20.9 ± 5.7 ^A^	29.8	Grass
#5	28.6 ± 8.2 ^A^	29.4	7.1 ± 5.5 ^B^	76.6	29.8 ± 7.8 ^A^	27.1	34.6 ± 7.1 ^A^	20.6	Tannin
#6	25.3 ± 10.0 ^AB^	39.5	18.1 ± 11.0 ^A^	59.6	31.7 ± 10.5 ^B^	31.9	24.9 ± 9.8 ^AB^	36.8	Legume
#7	28.0 ± 7.7 ^AB^	28.1	15.7 ± 11.1 ^A^	71.5	34.9 ± 13.9 ^B^	40.1	21.4 ± 8.9 ^AB^	45.3	Legume
#8	28.8 ± 8.0 ^A^	25.9	25.7 ± 3.7 ^A^	13.0	28.6 ± 9.5 ^A^	31.5	16.8 ± 2.6 ^B^	14.7	Essential oil
#9	38.4 ± 7.4 ^A^	21.4	13.9 ± 11.2 ^B^	80.7	26.1 ± 6.2 ^C^	27.7	21.6 ± 7.9 ^BCE^	36.6	Essential oil
#10	30.7 ± 6.7 ^AC^	18.4	5.8 ± 3.3 ^B^	55.2	37.6 ± 7.4 ^A^	19.0	25.9 ± 7.7 ^C^	28.9	Legume
#11	29.4 ± 5.4	19.2	25.9 ± 12.3	46.4	24.6 ± 8.6	35.2	20.1 ± 4.2	21.0	Essential oil
#12	28.3 ± 9.1 ^AC^	31.4	15.9 ± 9.0 ^B^	56.6	35.6 ± 6.5 ^C^	17.2	20.2 ± 7.9 ^AB^	33.8	Legume
#13	26.1 ± 4.6	19.3	23.0 ± 8.7	38.6	25.1 ± 5.2	23.3	25.9 ± 6.6	23.6	Essential oil
#14	25.1 ± 7.9 ^A^	29.6	16.3 ± 7.9 ^A^	49.0	39.1 ± 6.9 ^B^	17.5	19.5 ± 7.8 ^A^	37.9	Legume
#15	26.6 ± 12.8	47.1	26.5 ± 9.0	34.1	26.4 ± 9.6	32.7	20.6 ± 8.0	38.7	Essential oil
#16	21.7 ± 4.2 ^A^	19.7	18.8 ± 4.0 ^A^	20.5	41.6 ± 10.5 ^B^	24.2	17.8 ± 6.4 ^A^	36.1	Legume
#17	18.2 ± 3.5 ^A^	20.0	23.7 ± 8.0 ^A^	30.0	36.1 ± 7.8 ^B^	22.1	21.9 ± 5.1 ^A^	22.9	Legume
#18	22.5 ± 2.8 ^A^	13.1	21.6 ± 7.3 ^A^	31.7	25.8 ± 3.2 ^AB^	11.1	30.1 ± 7.6 ^B^	26.8	Tannin
#19	25.7 ± 10.0 ^AB^	39.5	24.5 ± 5.8 ^AB^	23.4	32.1 ± 5.6 ^A^	16.9	17.7 ± 6.7 ^B^	38.9	Legume
#20	28.1 ± 12.0	26.2	19.2 ± 4.2	23.1	25.8 ± 6.3	19.5	26.9 ± 11.3	32.2	Essential oil
#21	25.6 ± 3.9 ^A^	19.7	21.6 ± 5.6 ^A^	27.2	32.9 ± 7.2 ^B^	20.6	19.9 ± 6.0 ^A^	30.9	Legume
#22	23.4 ± 7.7	26.9	30.1 ± 12.8	39.4	22.8 ± 10.4	46.2	23.6 ± 8.1	34.7	Grass
#23	24.6 ± 6.1 ^AB^	26.7	24.6 ± 8.9 ^AB^	31.9	35.6 ± 13.2 ^A^	38.1	15.2 ± 6.6 ^B^	43.5	Legume

**Fig. 3:**
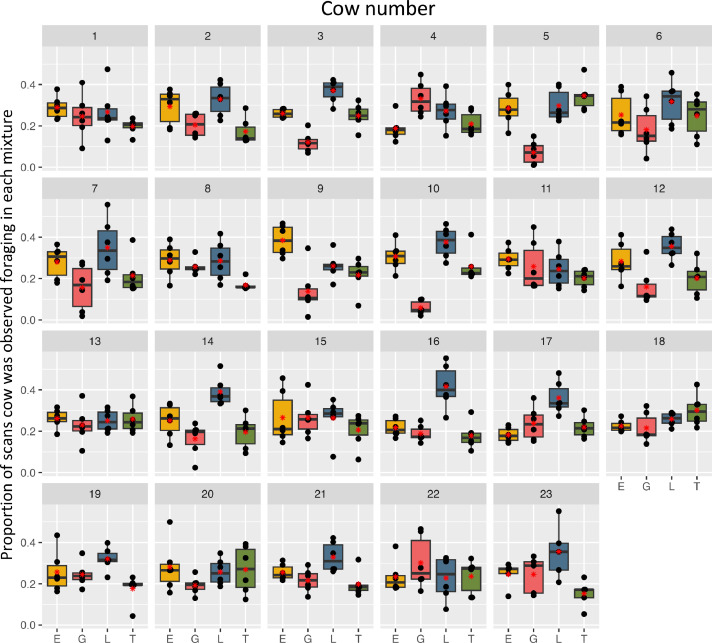
Proportion of scans the cows were observed foraging in the four plant mixtures across all grazing rotations. Each plot represents one individual. The graph is based on the average proportions of observations per treatment and rotation per individual (*n* = 6). The red asterisk indicates the mean value and the bold line the median. E Essential oil, G Grass, L Legume, T Tannin.

While the overall herd preference for mixture L could also be found for the majority of cows on individual level, almost half of the cows spent the highest proportion of scans foraging in other mixtures. Fig. 3 illustrates the proportion of scans, the individual animals spent foraging in the four plant mixtures, averaged over all rotations.

### 3.3 Consistency of individual preferences

The preferences of individual cows showed variation throughout the grazing season. The mixture they preferred in one rotation was not always the mixture they consumed most in the following rotation (Table 3). Various preference patterns were observed in individual cows. Most cows changed their foraging preferences throughout the grazing season, one cow did not show significant preferences in the majority of rotations, and one cow showed the same preference throughout the entire grazing season. Several cows could be observed preferring the L mixture for the first three rotations and then switching to other plant mixtures.

**Table 3:** Number of cows showing a preference for the respective mixture in each rotation (*N* = 22 to 23), based on the results of Chi-square tests for each cow within each rotation.

Rotation	Essential oil	Grass	Legume	Tannin
1	1	11	8	3
2	4	3	13	2
3	7	1	11	3
4	7	1	13	2
5	5	1	10	5
6	5	4	11	1

**Fig. 4:**
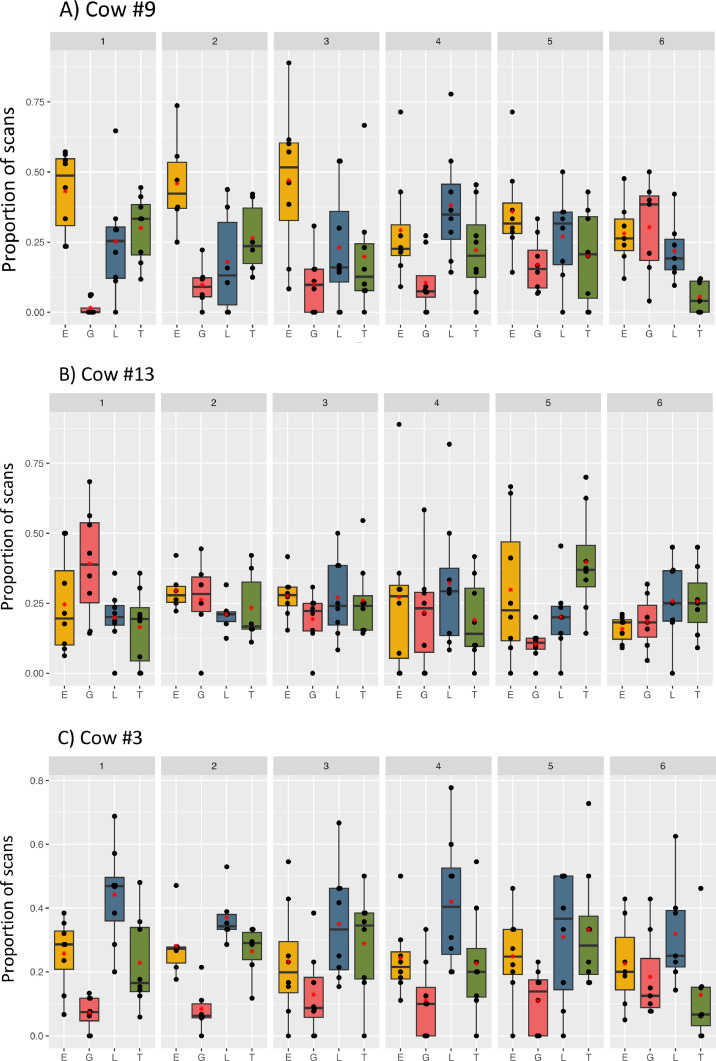
**A-C**. Selected examples of the foraging preferences of individual cows changing throughout the grazing season: Cow #9 (A), cow #13 (B) and cow #3 (C). The figures illustrate the proportions of scans each animal was observed foraging in each of the four plant mixtures during each grazing rotation (numbers 1 to 6). The graphs are based on the daily average proportions of observations per treatment and rotation per individual. The red asterisk indicates the mean value and the line the median. The whiskers of the boxplots illustrate the range of datapoints with each value depicted as a separate point. E Essential oil, G Grass, L Legume, T Tannin.

To illustrate examples of different foraging preferences, data from three selected individuals are presented in Fig. 4. Cow #9 spent most scans foraging in the E mixture for the first three rotations, but switched to different mixtures in the following three rotations (Fig. 4A). Cow #13 showed no consistent preference for any of the mixtures (Fig. 4B). In 4/6 rotations, the number of scans cow #13 spent feeding in the four mixtures did not differ significantly from an equal distribution (Chi-square test, *P* > 0.04). Cow #3 showed a preference for L in all rotations and the preference pattern was repeated in five out of six rotations (Fig. 4C).

To assess the consistency in the time spent grazing in the different mixtures for each cow, the coefficients of variance (cVar) were calculated (Table 2). The cVars were calculated based on the average proportion of scans each cow spent foraging in each mixture for each rotation, thus indicating the variance in preference between rotations. The cVars show very low values for some individuals and mixtures, indicating a very consistent foraging behaviour for the plant mixture (see e.g. 9 % in cow #3 foraging in E). For other individuals, higher values were found, indicating inconsistent foraging choices (see e.g., 81 % in cow #9).

## 4 Discussion

### 4.1 Foraging behaviour on herd level and individual level

On herd level, cows spent the highest average proportion of scans foraging in the L mixture. However, individual cows showed preferences that deviated from the herd average and individual preferences showed temporal variation throughout the grazing season. For all but two cows, the preference for the plant mixtures shifted across rotations. This variability highlights that herd-level averages may not always reflect individual behaviors or needs. As a general finding it has to be remarked that all cows spent time foraging in all mixtures, consuming all visible plant species. This demonstrates that the implementation of spatially segregated multispecies swards on existing pastures may be a feasible option to introduce dietary variation, which the cows utilized. Overall, the cows’ preferences deviated not drastically from a random pattern, as they spent time foraging in all mixtures, supporting the concept of 'partial preference' (Parsons et al., 1994; Poli et al., 2006; Rutter, 2010; Rutter et al., 2004). Therefore, when discussing the preferences mentioned in the results section, it has to be considered that these were only partial and that all cows grazed on all offered mixtures in all rotations.

On a systemic level, there are broad general laws governing forage choice, e.g., plants with a high nutritive value are preferred over plants with a low nutritive value (Baumont et al., 2000), as stated by the optimal foraging theory. Additionally, there may be other factors that determine the “finetuning” of forage choice. These may include plant properties (structural, chemical composition) (C.M. Pauler et al., 2020b) and their digestibility (Meisser et al., 2014), their content of PSMs and plant primary metabolites (PPMs) (Villalba et al., 2015), plant community properties (species abundance, spatial allocation and vertical arrangement of plants) (Agnusdei & Mazzanti, 2001; Carpino et al., 2003; Meisser et al., 2014) and the landscape context (slope, allocation of water trough, etc.) (Bailey et al., 1996; Rivero et al., 2021). The overall herd-level preference was fairly consistent across rotations. In four out of six rotations, the preference pattern *L* > *E* > *T* > *G* was observed. On herd-level and in all rotations, the highest proportion of scans was spent foraging in L, which had the highest proportion of legumes, the highest contents in energy and protein, and the highest overall yield. This finding supports a partial preference of cattle for legume species as found in dietary choice experiments on pasture or semi-natural grasslands (Dumont et al., 2007; Francis et al., 2006; Searle & Shipley, 2008). A grazing trial with different cattle breeds by Dumont et al. (2007) observed a bite selection dominated by legumes or forbs for heifers and steers.

The E mixture was designed to have a high content in essential oils. However, the botanical community developed differently and, compared to the other mixtures, it had intermediate values in crude protein (CP), energy (NEL), and fiber (ADF). White clover established itself in the plots of the E mixture. This increased the proportion of legumes and thus protein, which may have increased the attractiveness of the E mixture. On herd-level, the T mixture ranked third, possibly due to the lowest energy content (NEL) and the highest values of ADL, and low OM and crude fat values. The G mixture was least preferred, likely because of its high fiber content (NDF), low protein content, and moderate energy content (NEL). In addition to the pasture forage, cows received grass-based hay ad libitum in the stable. During rotation 1 to 3, they were also fed some grass silage. The composition and consumed quantity of these additional feedstuffs may have influenced the animals’ choices on pasture. For example, the hay had an average NDF content of 50 %, which is higher than any of the swards. This may have resulted in the cows choosing the mixture rich in protein, rather than the mixture rich in fibre.

Seasonal variations in botanical and nutrient composition, weather, and climate may also explain the fluctuating preferences. The phenological stage of the plants and environmental factors related to the season such as high light intensity, extreme temperatures, or water stress may influence the CT-content in plants (Kälber et al., 2014; Rodríguez-Hernández et al., 2023). The deviating foraging patterns in the first and last rotation at the herd level may partly be explained by the nutrient composition of the pasture. One possible explanation for the fact that the cows spent more time foraging in the G mixture in rotations 1 and 6 compared to rotations 2–5 could be the relatively low ADL content in both rotations. A further reason for a changed feed intake of the different plant mixtures over the grazing season may be a change in palatability, due to changing contents of PSMs such as tannins, or physical attributes as increasing lignification (Rochfort et al., 2008). Furthermore, there were diurnal changes in the grazing times throughout the season. During the hot summer months (rotation 2 to 4) the herd was given access to pasture in the early morning or the late evening. An effect of daytime on foraging behaviour was observed in some studies. For example, Rutter et al. (2004) found that cows preferred white clover in the morning, and ryegrass in the afternoon. The data presented in this study were generated during a single grazing season and one can only speculate, if and how the herd preference would change in response to different climatic conditions between years, which would affect pasture properties.

### 4.2 Individual preferences

No animal behaved in a strictly selective and consistent manner, at least not in the sense that the individual always showed the same preference pattern over the entire grazing season. Some animals behaved fairly consistently over all rotations, others showed different preferences throughout the grazing season and a third category did not show significant preferences for any mixture. These different preference patterns could be classified into the three identified foraging types (1) selective-consistent, (2) selective-inconsistent, and (3) non-selective-consistent. The possible reasons for individual differences in foraging behaviours are manifold. Neave et al. (2018) identify three key personality traits that may influence foraging behaviour: exploration, fear/reactivity, and sociability. The position of a cow on pasture is thus not only determined by foraging preference, but also by social interactions and relations, herd hierarchy and dynamics (Neave et al., 2018). Since dairy cattle are social animals, the behavior of other herd members may influence their foraging choices (Forbes & Kyriazakis, 1995). During grazing, a majority of the animals could be observed moving collectively as a herd across the experimental site, which may have influenced the location in which some animals where grazing. Some cows appeared to have affiliative relationships, while others showed aversive behaviour towards herd members. Therefore, it has to be assumed that social interactions had an influence on foraging location. However, the replicated block design of the pasture should have eliminated these effects to a large extent.

Differences in individual preferences may be also due to a variety of factors such as the morphological and physiological state of the animal (Gregorini et al., 2008; Villalba & Provenza, 2007), its current nutritional state (Mangel & Clark, 1986; Scott & Provenza, 1999), the composition of its ruminal microflora (Gregorini et al., 2008) and breed (Dumont et al., 2007; Hessle et al., 2014, 2008; C.M. Pauler et al., 2020a). Recent research in biopsychology also stresses the effect of hedonic aspects such as pleasure (Villalba et al., 2015) and ‘reward learning’ (Dalton & Finlayson, 2013) to potentially influence the foraging behaviour. In addition to genetic predispositions, which may influence feed choice (Meagher et al., 2017), the latter is also influenced by previous experience (Lyman et al., 2011; Villalba et al., 2006a). Past foraging experiences made *in utero* (Wiedmeier et al., 2012) early in life, or during the recent past (Villalba et al., 2015) may affect the foraging decisions. Some of the cows in the herd grew up on the experimental farm, while others had been raised on a different farm. This may have resulted in different exposure to pasture plants, resulting in different preferences.

The nutritional state of the individual also influences preferences (Provenza et al., 2003). Depending on which nutrients, vitamins, or minerals are currently deficient, different plants are consumed (Neave et al., 2018). In addition, the composition of the microflora in the digestive tract seems to play a role in which substances currently have a utility for the organism (Gregorini et al., 2008). To better understand the reasons for variation between individuals, future research under more controlled experimental conditions may help identifying factors determining foraging decisions.

### 4.3 Relevance for agricultural practice

The fact, that the cows grazed on all of the spatially segregated swards shows that this concept of pasture design may be of interest for the agricultural community. Many studies claim positive environmental effects of mixed swards, such as carbon sequestration or increased drought resistance (Isbell et al., 2015; Lüscher et al., 2022; Skinner & Dell, 2016) or nutritive yield effects (Schaub et al., 2020). In the context of climate adaptation, designing pastures with mixtures that peak at different times may help maintain stable yields during climatic extremes (Delaby et al., 2020).

Allowing selective foraging may improve animal welfare through the ability to express natural species-specific behaviour, as well as enhance intake and productivity. The consumption of tannin-containing plants has been linked to improved protein utilization (Kapp-Bitter et al., 2023, 2021; Min & Hart, 2003), increased milk yields (Pembleton et al., 2016; Roca-Fernández et al., 2016), and faster growth rates (Handcock et al., 2015). Furthermore, allowing ruminants to select from a variety of pasture plants may improve their health. The potential medicinal effects of plants rich in PSM includes reduced parasite infestation (Barry, 1998; Min & Hart, 2003) or the prevention of pasture bloat (McMahon et al., 2000; Mueller‐Harvey, 2006; Waghorn & Jones, 1989). Therefore, the establishment of multi-species pastures may be an inexpensive measure to increase animal health and wellbeing and to reduce veterinary costs.

## 5 Conclusion

The cows grazed on all four spatially segregated multispecies swards. On herd level, the cows showed a partial preference for the swards rich in legumes, but preferences of individual animals differed from the herd average. While the reasons for individual differences in preference remain unknown, the results demonstrate that cows make use of pasture diversity. Allowing them to select from a variety of pasture plants may improve their welfare and give them the option for self-medication or balancing of rumen processes. This study is an initial step to understanding individual foraging behavior on biodiverse pastures. Further research is needed to investigate the mechanisms driving individual variation.

## Data availability

The data generated during this study are available from the corresponding author upon reasonable request.

## Animal welfare statement

The experimental protocol was approved by the Animal Health and Welfare commission of the Canton Aargau, Switzerland (National license Nr. 34390).

## Funding

We gratefully acknowledge the generous financial support of the Foundation Edith Maryon, Basel.

## CRediT authorship contribution statement

**Mira Hesselmann:** Writing – review & editing, Writing – original draft, Visualization, Methodology, Investigation, Formal analysis, Data curation. **Sarah Thorne:** Writing – review & editing, Methodology, Investigation. **Amarante Vitra:** Writing – review & editing, Methodology, Investigation, Formal analysis, Conceptualization. **Andrea K. Steiner:** Writing – review & editing, Methodology, Investigation. **Florian Leiber:** Writing – review & editing, Supervision, Funding acquisition, Conceptualization. **Marie T. Dittmann:** Writing – review & editing, Writing – original draft, Visualization, Supervision, Project administration, Methodology, Investigation, Formal analysis, Conceptualization.

## Declaration of competing interest

The authors declare that they have no known competing financial interests or personal relationships that could have appeared to influence the work reported in this paper.
